# Restoration of Spatially Variant Blurred Images with Wide-Field Telescope Based on Deep Learning

**DOI:** 10.3390/s23073745

**Published:** 2023-04-04

**Authors:** Yingmei Tian, Jianli Wang, Junchi Liu, Xiangji Guo

**Affiliations:** 1 Changchun Institute of Optics, Fine Mechanics and Physics (CIOMP), Chinese Academy of Sciences, Changchun 130033, China; 2 College of Optoelectronics, University of Chinese Academy of Sciences (UCAS), Beijing 100049, China

**Keywords:** image restoration, wide-field astronomical image, spatially variant deblur, deep learning

## Abstract

The wide-field telescope is a research hotspot in the field of aerospace. Increasing the field of view of the telescope can expand the observation range and enhance the observation ability. However, a wide field will cause some spatially variant optical aberrations, which makes it difficult to obtain stellar information accurately from astronomical images. Therefore, we propose a network for restoring wide-field astronomical images by correcting optical aberrations, called ASANet. Based on the encoder–decoder structure, ASANet improves the original feature extraction module, adds skip connection, and adds a self-attention module. With these methods, we enhanced the capability to focus on the image globally and retain the shallow features in the original image to the maximum extent. At the same time, we created a new dataset of astronomical aberration images as the input of ASANet. Finally, we carried out some experiments to prove that the structure of ASANet is meaningful from two aspects of the image restoration effect and quality evaluation index. According to the experimental results, compared with other deblur networks, the PSNR and SSIM of ASANet are improved by about 0.5 and 0.02 db, respectively.

## 1. Introduction

With the development of the times and science and technology, space has gradually become an important position of scientific and technological competition among countries. Astronomical observation provides an important information basis for this purpose, and the telescope is an important instrument for astronomical observation. Therefore, people begin to pay attention to improving the observation ability of astronomical telescopes, and increasing the field of view is the current hot research direction. Increasing the field of view can provide the telescope with wider sky coverage [1] and enhance the observation capability. However, increasing the field of view of the telescope means that multiple SCMOS or CCD need to be splicing, which leads to the unevenness of the detector plane, the complexity of the optical design [2], and the introduction of additional refraction elements.

For wide-field astronomical telescopes, different factors, such as the dispersion of refracting materials, alignment error of components in the observation process, and uneven optical surface, will produce more complex and serious aberrations than ordinary mirror telescopes [3]. These aberrations will lead to the spatiotemporal variation of the optical system quality of the telescope, and introduce the varying point diffusion function (PSF) in different parts of different images [4], resulting in distorted astronomical images that can only be obtained through the telescope, reducing the effective resolution of image weak objects [5], and losing effective information. In order to eliminate these effects, post-processing of astronomical images has become a necessary task.

One of the most common methods is considering a piecewise invariant PSF [6]. The image is thus segmented into sub-images where the blur is assumed to be invariant. Blind deconvolution methods are then applied to each of these sub-images separately. One of the classic segmental PATCH [7] algorithms has been applied to the telescope image processing of adaptive optics systems to improve the results of astrometry and photometry. However, these methods lead to decomposition artifacts on the reconstructed image [8].

Later methods consider a smoothly varying blur where the space-variant PSF is modeled by a combination of space-invariant ones [9,10]. These models differ in the way they interpolate spatially invariant PSFs. However, due to the complex aberrations and initial PSF parameters in wide-field telescope images, this method is not practical in wide-field astronomical image restoration.

Meanwhile, with the development of neural networks, deep learning has gradually come into the limelight. The parallel structure of neural networks improves the operational efficiency and computational power of the networks, enabling them to adapt to the local nature of the problem and handle more complex mappings. As a result, they are increasingly applied to spatially variant blurred image processing, such as using traditional methods in combination with deep-learning methods. Schuler et al. [11] proposed to learn the prior of an image by employing a CNN, and then use prior constraints for blur kernel estimation and finally image deblurring. Yan et al. [12] proposed to use deep learning for estimating the blur kernel, in reconstructing clear images using adaptive likelihood probability log-expectation. Sun et al. [13] proposed an image deblurring method based on CNN and Markov random field. The method focuses on estimating the probability distribution of blurred image blocks by CNN, further extending the candidate set of motion kernels predicted by CNN, and finally using Markov random field model to derive a dense nonuniform motion blur field to enhance the motion smoothing. These methods mentioned above do not take an end-to-end training approach, and thus the image deblurring process requires a traditional non-blind deblurring step, resulting in a long, time-consuming process.

Therefore, many end-to-end methods have also been applied in the field of image restoration. For example, Zhang et al. used a network combining CNN and RNN to achieve end-to-end recovery of motion blurred images [14]. Yuan et al. proposed a dynamic scene deblurring method based on optical flow-guided training and spatial variable inverse fold product [15]. Nah proposed a multiscale neural network [16] for the hierarchical recovery of Gaussian blur [17]. Jung et al. [18] proposed an advanced U-Net model based on global and local residual learning and has an unmatched performance of previous methods in recovering complex degraded images. In the same year, Jin et al. [19] proposed an image recovery algorithm based on GAN and multi-scale feature fusion, which successfully generated more realistic recovered images while improving the image recovery accuracy. Sainandan et al. [20] used a generative adversarial model to recover real motion blurred images and proposed to calculate the image perceptual loss using the feature map of VGGNet model to solve the image The problem of recovering structural details was solved by proposing to compute image perceptual loss using the feature maps of the VGGNet model.

Deep learning has good applications in the field of astronomical image restoration. In 2017, Flamery [21] used CNN for astronomical image reconstruction; Schaweinski et al. [22] used deep neural networks (DNN) that can handle classical galaxy image inverse fold products well. In 2018, Sánchez et al. [23] used deep learning to improve the galaxy morphology of SDSS. In 2019, Sureau [24] used a U-NET deep neural network (DNN) architecture to learn parameters suitable for galaxy image processing in a supervised setting and investigated two anti-folding strategies based on Tikhonov and AMDD. Akhaury [25] used the Tikhonov closed form of the anti-fold product and used Learnlet to anti-fold galaxy images. In 2021, Buncher et al. [26] used GAN for galaxy image reconstruction. In 2022, Nammour added a new shape constraint called Shapenet [27] to Tikhonet to make it more effective in the anti-folding of galaxy images.

However, the current network structure of astronomical image restoration is mostly focused on galaxy images, and few people pay attention to star image restoration.

In astronomical missions, stars can be regarded as almost perfect point sources. Measuring the position and brightness of stars can be used as a reference point for analysis, calibration, and scientific research. However, in a real astronomical image, stars occupy a very small proportion of the entire image, and the distribution is not regular. There may be almost no stars on one side but a dense distribution on the other side, or there may be a dense distribution of stars throughout the entire image. This leads to the possibility of crosstalk between stars, which cannot easily be regarded as separate entities. Especially in images taken by wide-field telescopes, varying optical aberrations can lead to more pronounced differences in regional PSFs.

Due to the above reasons, it is particularly difficult for wide-field telescopes to obtain the PSF prior information in the captured images, which can be avoided by end-to-end networks in deep learning.

Our proposed network is inspired by U-Net [28]. U-Net is a classical algorithm proposed by Ronneberger et al. in 2015, which has a wide range of applications in the field of image segmentation, and people still keep innovating on it, such as MultiResU-Net proposed in 2019 [29], DC-U-Net in 2020 [30], etc. Gradually, U-Net has been applied to other fields as well. In 2020, Dong et al. [31] proposed a U-Net-based multi-scale network for image defogging. In 2021, Cho et al. [32] proposed MIMO-U-Net for implementing image deblurring with multi-scale inputs.

Therefore, in this paper, we proposed a deep-learning network for the recovery of images captured by ground-based wide-field telescopes. We adopted the common encoder-jie decoder structure, set up a U-shaped structure similar to U-Net, added skip connections between the encoder and decoder, updated the feature extraction module, and added a self-attention module to the internal feature extraction part for connecting the global and enhancing the feature extraction capability.

The main contributions of this work are as follows:

- We proposed a network structure for star images, which enhances the feature ex-traction capability of the network by adding a self-attention mechanism;
- Through the correspondence between Zernike polynomial and optical aberration, we created a dataset composed of optical aberration images, which can be used to imitate star images taken by wide-field astronomical telescopes;
- Through experiments, our method had a good restoration effect on both the training set image and the real image. Compared with the existing methods, our method had some improvement in PSNR and SSIM.

The organizational structure of the full text is as follows. In Section 2, we describe the ASANet architecture and introduce how the self-attention module works, and in Section 3, we describe how the dataset is created and briefly explain the selection of training strategies. In Section 4, we conduct some experiments, recorded the selection of some hyperparameters, and compare the restoration effects of other methods, proving the feasibility and effectiveness of ASANet. In Section 5 and Section 6, we provide an analytical discussion of the experimental results and a final summary.

## 2. Network Architecture

Our approach is based on images obtained in the star pattern, where stars appear as dots in the image. Unlike traditional images, each star in these images varies in size and brightness, some may occupy only a few pixels, the distribution of stars may be sparse or dense, and there is overlap between stars. These features are part of the reason for detail loss in the multi-sampling process of convolution and pooling. The information in the image cannot be completely restored even after the feature extraction after the subsequent up-sampling. Therefore, we should strengthen the use of feature mapping information in front of the architecture to preserve feature details, which draws inspiration from U-Net.

**Figure 1:**
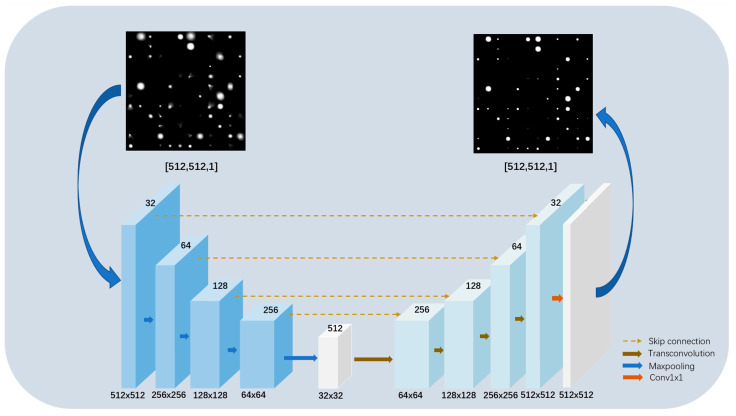
The external main structure of the proposed network. The blue cube on the left represents the encoder structure, and the cyan cube on the right represents the decoder structure.

Therefore, we propose a network for feature extraction and recovery of input astronomical images, also based on encoder–decoder mechanisms. As shown in Figure 1, the blue cube on the left represents the encoder used to compress the image and extract features, and the cyan cube on the right represents the decoder used to recover the image. Since autoencoder structures typically have a “thick on both sides and thin in the middle” shape, the middle position can be used to extract the most prominent features. Therefore, the white box at the bottom of Figure 1 contains only 3 × 3 convolution layers for feature extraction. At the end of the decoder is the 1 × 1 convolution layer, which is used to adjust the output image to an ideal image of the same shape as the input image.

**Figure 2:**
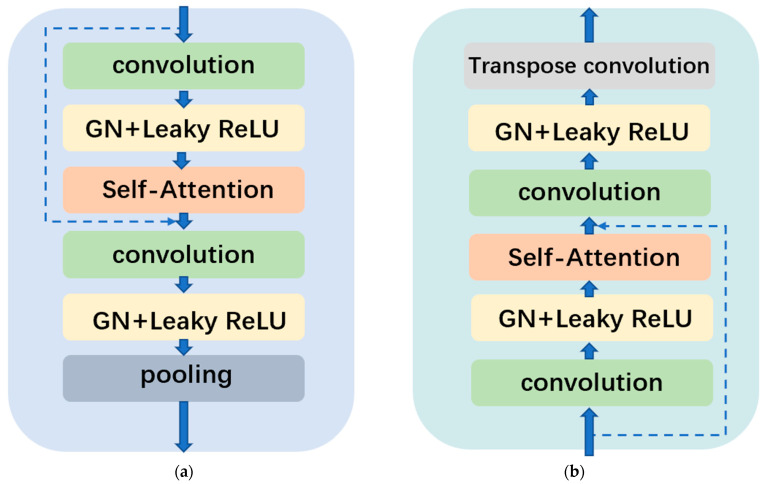
(**a**) Encoder module, using maxpooling to realize down sampling; (**b**) decoder module, using transconvolution to realize up-sampling.

The functional implementation of this network depends on the encoder and decoder, whose internal structure is shown in Figure 2.

As shown in Figure 2, inside each layer of the encoder, we set two convolution layers as the main bearers of feature extraction, and the convolution kernel size is 3 × 3.After each convolution, there are the normalization layer and the excitation layer. We used group normalization (hereinafter called “GN”) and the nonlinear Leaky ReLU layer, respectively, replacing the common BN (batch normalization) + ReLU.

This is because BN is used to normalize the features extracted from the corresponding channel in batch, and the dimension is [N, H, W]. Although it has a good normalization effect, it is overdependent on the selection of batch size. Once batch size is too small (such as batch size = 2, 4 or even sometimes batch size = 8), the network performance may deteriorate. If we choose a large batch size for the field of image processing, the size of the input target is often large, which will greatly increase the amount of calculation and put forward higher requirements for hardware configuration.

However, GN is different. Firstly, it divides it into multiple groups based on the channel dimension, adjusts the dimension of features from [N, C, H, W] to [N, G, C//G, H, W], and then normalizes each group. The normalized dimension is [C//G, H, W]. Therefore, using GN instead of BN can still speed up the training and convergence speed, but remove the dependence on batch size and effectively enhance the feature extraction ability.

As for using Leaky ReLU instead of ReLU, it is because Leaky ReLU gives a non-zero slope to a negative value, unlike ReLU, which avoids the “dead neuron” by setting its output to zero on negative input values.

At the end of each encoder, the maxpooling module is used to realize image downsampling. In the decoder, this module is replaced with deconvolution to realize upsampling, which is also the biggest difference between the decoder and the encoder structure. Most importantly, we introduced the self-attention module, which is placed between the two layers of convolution, and add the forward join, which, together with the second layer GN, can mitigate the possible effects of gradient disappearance and gradient dispersion.

However, the representation ability of the network is still limited by the kernel size of the neighborhood. Even if the receptive field gradually increases in the later period, it can only pay attention to the local area and ignore the contribution of other parts of the global area (such as pixels far away) to the current area. To solve this problem, we introduced the self-attention mechanism [33].

**Figure 3:**
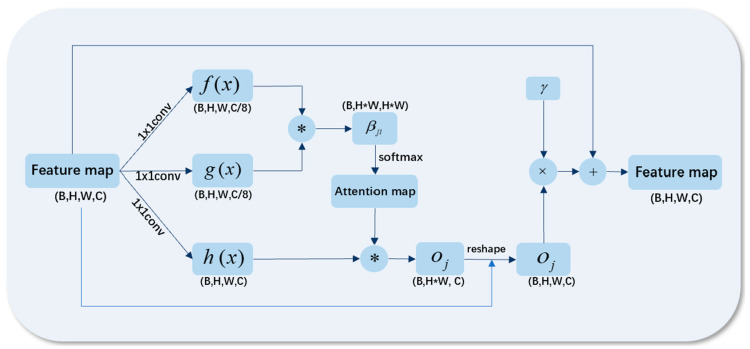
Schematic diagram of self-attention mechanism.

The self-attention mechanism is a variant of the attention mechanism that reduces its dependence on external information and is better at capturing the internal relevance of data or features. In the field of image processing, the self-attention mechanism learns the relationship between one pixel and pixels in all other locations, and uses the features of all locations to help generate the details of the picture. The working principle is shown in Figure 3.

In the self-attention module, we took the feature map output by the convolution layer as input x, and carried out linear mapping (usually referring to 1 × 1 convolution) of x to obtain three feature spaces, f, g, and h. Then we used them to calculate the weight of self-attention:

(1) $$s_{ij}=f{\left(x_{i}\right)}^{T}g(x_{j})$$

(2) $$\beta_{j,i}=\frac{\exp\left(s_{ij}\right)}{\sum_{i=1}^{N}\exp\left(s_{ij}\right)}$$

where $\beta_{j,i}$ is used to represent the relationship weight of the $i^{th}$ position to the generated $j^{th}$ region, also known as the attention map, and N is the number of feature positions.

We multiplied h with $\beta_{j,i}$ adjusted by Softmax to attain the output result of self-attention layer:

(3) $$o_{j}=\sum_{i=1}^{N}\beta_{j,i}h\left(x_{i}\right)$$

(4) $$O_{j}=v\left(o_{j}\right)$$

where v(x) represents 1 × 1 conv, which is used to adjust the shape of the output result to be consistent with the input feature map.

We multiplied the above results by a learnable coefficient γ and added the original feature map to obtain a new feature map for a new round of feature extraction:

(5) $$y_{i}=x_{i}+\gamma O_{i}$$

After increasing self-attention, we mainly relied on neighborhood features in the initial stage of network training, and then gradually increased the weight of dependence on distant regions, which solved the problem of long-range dependencies of common convolutional structures.

Compared with other methods, ASANet is able to consider the overall situation and focus on the key points, and fully take into account every “independent” star point, so that they are not subject to cross-talk between neighboring stars, so as to achieve good restoration effect.

## 3. Dataset Generation and Learning Details

Most of the common astronomical image restoration research aims to realize the morphological classification of galaxies. The object of this kind of restoration is the galaxy image with a certain contour and structure. The blur and aberration usually come from the atmospheric distortion caused by atmospheric turbulence rather than the error of the optical system itself. Thus, in such experiments, ideal contrast images can be obtained with space-based telescopes, without atmospheric interference. For example, Utsav Akhaury et al. extracted HST cuts measured by CANDELS [34] from F606W filters (V-band) and destroyed these images to simulate their blurred noise version; Sureau et al. directly uses the HST image without atmospheric turbulence to convolve the Euclidean PSF with known variations to form a certain spatial variation ambiguity.

However, what we needed to recover was the astronomical image taken by the ground-based wide-field survey telescope. The optical aberration mainly comes from the unflatness of the wide-field detector and the optical system error rather than the atmospheric turbulence. The systematic error could not be eliminated, so it was difficult to obtain the ideal real control image, which had to be realized by simulation.

### 3.1. Zernike Polynomial

Aberration is a kind of imaging defect in the optical system, which can be decomposed into a linear combination of orthogonal polynomials. Since the orthogonality of Zernike polynomial in a circle has the characteristics of inverse transformation and minimum information redundancy of the described image, and each order mode can correspond to Seidel aberrations in optical design (such as defocus, astigmatism, coma, etc.), in this paper, we chose Zernike polynomial as the basis function of aberration description:

(6) $$\varphi (\rho ,\theta )=\sum_{i=1}^{n}a_{i}Z_{i}(\rho ,\theta )$$

Here, φ(ρ,θ) is the phase distribution on the pupil plane, n is the highest order of Zernike polynomial, $Z_{i}$ and $a_{i}$ represent the ith term polynomial and its coefficients, respectively, ρ is the polar radius, and θ is the polar angle.

Zernike polynomials usually assume that the maximum number of terms is 35, of which the first eight terms are defined as low-order aberrations, followed by higher-order aberrations. In this article, we only used the first 15 Zernike terms for aberration coupling.

### 3.2. Aberration Image Generation

**Figure 4:**
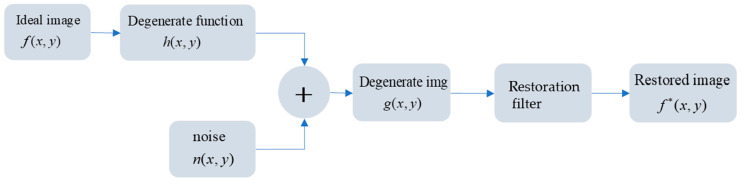
Process of image degradation and restoration.

Generally speaking, image degradation is to transform an image from an ideal image into an actual defect image we see, while image restoration is on the contrary. The whole process can be shown in Figure 4 below.

Here, we expressed the ideal image with f(x,y) and the degraded image with g(x,y). The degradation process can be divided into the degradation function h(x,y) (usually known as point spread function, PSF) and the noise loaded on the image n, with ∗ representing the convolution process. Then, the image degradation model can be expressed as:

(7) g(x,y)=h(x,y)∗f(x,y)+n(x,y)

To put it simply, the convolution of the original image and PSF is the main factor of image degradation. Therefore, to simulate an aberration image, the ideal original star should be selected first, and then the appropriate PSF should be convolved with it to generate an aberration image.

For wide-field telescopes, due to the change of incident angle, the image presented by the edge field of view and the center field of view will be significantly different. Therefore, we manually cut out circular stars with clear outlines and saturated brightness from the central region of the real image as ideal stars for subsequent generation of single optical aberrations.

According to Fourier optics, the general imaging process can be regarded as a low-pass filtering operation on the spectrum of an object. The low-pass filter is called the transfer function in optics, its Fourier transform is called the shock function, and the square of the modulus of the shock function is called the point spread function (PSF). In short, PSF is the modular square of the Fourier transform of the wavefront function at the pupil so that the Zernike polynomial which corresponds with Seidel aberration one by one could be chosen as a transfer function to generate corresponding PSF.

**Figure 5:**
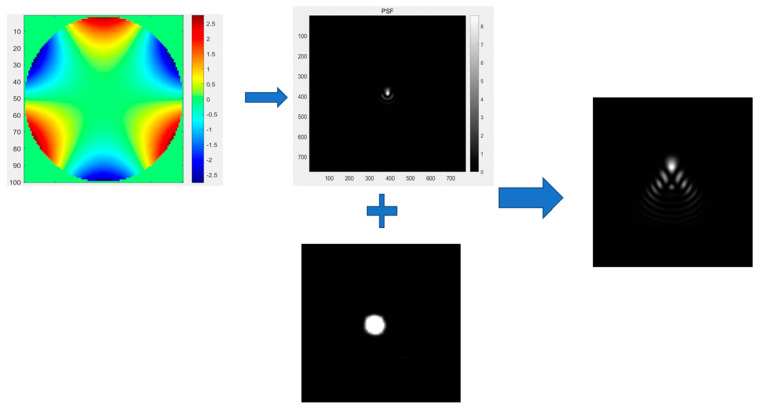
Use the generated PSF to convolute with the selected ideal star to obtain the image of optical aberration.

According to the process of image degradation, we can convolve the original star with PSF to attain an aberration image. In Figure 5, we illustrated this process. We used the seventh term of the Zernike polynomial to fit PSF, and intercepted the star from the real astronomical image as the ideal point target, and finally convolved the two to obtain the comet image.

The distribution of star points in the real star map is random and disorderly, and multiple star points may overlap and block each other. To achieve this effect, we used 30,000 single star point aberrations and blank images filled with pure black to carry out random, multiple, and multi-level overlapping and splicing, to ensure that the number and distribution of star points in each image are irregular, and the whole dataset should have authenticity and specificity at the same time. Finally, a labeled dataset containing 500 spatially variant aberration images was generated.

### 3.3. Network Learning Details

In the training process, we used a dataset image with a resolution of 512 × 512 as the training input and set the batch size to 8. The activation function was chosen as Leaky ReLU, He Initialization can ensure that the information can flow effectively during the forward and backward propagation, so that the variances of the input signals of different layers were approximately equal, and therefore more suitable for initializing the network parameters in this network model. L2 loss was used as the loss function and a combination of Adagrad and SGD was used for network optimization. The initial learning rate was set to 0.01. According to our experiments, 500 epochs were sufficient for the network to converge.

## 4. Experiments

In this section, we first introduced the evaluation metrics used to judge the quality of image restoration. Secondly, different hyperparameters were set for training to select the optimal training strategy. Then, the ablation study was carried out to verify that the increased self-attention structure was meaningful. Finally, we used different methods to restore the real astronomical image, and verified the feasibility of the algorithm by comparison.

Our experiments were conducted on a computer with 16 GB of RAM, an Intel Core i7 8700 K, a 3.6 GHz processor, and an Nvidia 1080ti GPU. The network architecture was implemented in TensorFlow2.0 and Python 3.6.

### 4.1. Evaluation Metrics

Image quality evaluation methods can be divided into subjective evaluation methods and objective evaluation methods. Subjective methods are based on human judgment and may not require reference to images. For image restoration, most of the existing methods are based on the full reference evaluation in the objective evaluation methods. The ideal image was selected as the reference, the difference between the image to be evaluated and the reference image was compared, and the distortion degree was evaluated.

In this paper, we chose PSNR and SSIM, which are widely used in the image field.

#### 4.1.1. PSNR

MSE (mean square error) and PSNR (peak signal-to-noise ratio) are common quality assessment methods based on pixel statistics. They measure the quality of the image to be evaluated from a statistical point of view by calculating the difference between the gray value of the corresponding pixel of the image to be evaluated and the reference image. The definition formula is as follows:

(8) $$MSE=\frac{1}{m\times n}\sum_{i=0}^{m-1}\sum_{j=0}^{n-1}{\left[I(i,j)-K(i,j)\right]}^{2}$$

where I(i,j) and K(i,j), respectively, represent represents the grayscale value of each pixel in the original and the restored image, m and n are the length and width of the image.

(9) $$PSNR=10\log_{10}\frac{{\;255}^{2}}{MSE}$$

#### 4.1.2. SSIM

PSNR and MSE measure image quality by calculating the global size of the pixel error between the image to be evaluated and the reference image, ignoring some visual features contained in the image content, especially the local structure in image formation. Therefore, we introduced an evaluation index SSIM (structural similarity) based on structural information.

SSIM evaluates the similarity of the two images through brightness l(x,y), contrast c(x,y), and structure s(x,y). These three dimensions form a complementary relationship and form a description of image quality under the common constraints of the three dimensions. Therefore, SSIM can be expressed as:

(10) $$SSIM(x,y)={\left[l(x,y)\right]}^{\alpha }{\left[c(x,y)\right]}^{\beta }{\left[s(x,y)\right]}^{\gamma }$$

where α, β, γ are used to adjust the weight of three parts. In actual calculation, we usually chose α=β=γ=1.

After expanding l(x,y), c(x,y) and s(x,y), respectively:

(11) $$l(x,y)=\frac{2\mu_{x}\mu_{y}}{\mu_{x}^{2}+\mu_{y}^{2}}\overset{\;\mathrm{stabilization}}{=}\frac{2\mu_{x}\mu_{y}+C_{1}}{\mu_{x}^{2}+\mu_{y}^{2}+C_{1}}$$

(12) $$c(x,y)=\frac{2\sigma_{x}\sigma_{y}}{\sigma_{x}^{2}+\sigma_{y}^{2}}\overset{\;\mathrm{stabilization}}{=}\frac{2\sigma_{x}\sigma_{y}+C_{2}}{\sigma_{x}^{2}+\sigma_{y}^{2}+C_{2}}$$

(13) $$s(x,y)=\frac{\sigma_{xy}}{\sigma_{x}\sigma_{y}}\overset{\;\mathrm{stabilization}}{=}\frac{\sigma_{xy}+C_{3}}{\sigma_{x}\sigma_{y}+C_{3}}$$

where $\mu_{x}$ and $\mu_{y}$ represent the standard deviation of images x and y, $\sigma_{x}$ and $\sigma_{y}$ represent the sum of the variances of x and y, $\sigma_{xy}$ is the covariance of x and y, and $\;C_{1}\;$, $\;C_{2}\;$, and $\;C_{3}\;$ are constants for making the calculation more stable.

Then, SSIM can be expressed as follows:

(14) $$SSIM(x,y)=\frac{\left(2\mu_{x}\mu_{y}+C_{1}\right)\left(2\sigma_{xy}+C_{2}\right)}{\left(\mu_{x}^{2}+\mu_{y}^{2}+C_{1}\right)\left(\sigma_{x}^{2}+\sigma_{y}^{2}+C_{2}\right)}$$

### 4.2. Training Strategy Selection

In the learning process of the network, the input image should be preprocessed to adjust the resolution of the image, so as to improve the computing speed and learning effect of the network. Hyperparameters such as loss function and optimizer have a significant impact on the training effect of the network and should be selected carefully.

#### 4.2.1. Comparison of Images with Different Resolutions

Increasing the image resolution can improve the performance difference between stars and other regions, so that the convolutional neural network can learn more detailed features. Reducing the image resolution can reduce the computing cost to the greatest extent and speed up the network operation. Considering the limitation of the computing power of the server, we send the image resolution of 512 × 512 and 256 × 256 to the network, respectively, as a test. To ensure fairness, the image with different resolutions is uniformly adjusted back to 512 × 512 after the network output.

**Figure 6:**
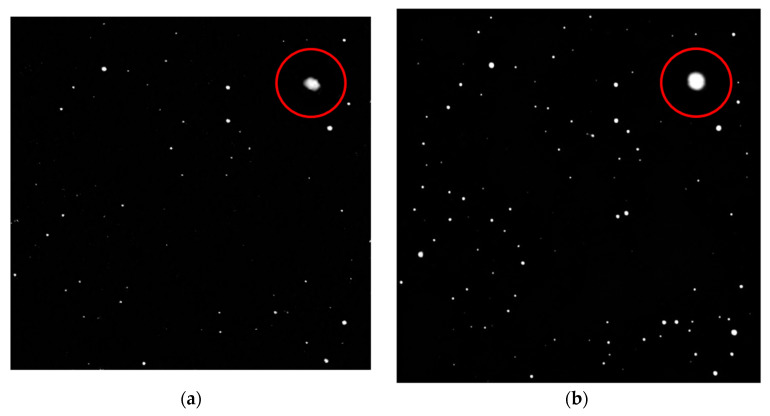
(**a**) The training results after the image with a resolution of 256 × 256 is input into the network; (**b**) the training results after the image with a resolution of 512 × 512 is input into the network.

As can be seen in Figure 6, the 256 × 256 image on the left, restored to 512 × 512 size via the network, has weaker star energy, marked jagged edges, and far fewer stars than the one on the right. This is because the resolution of the input image is too small to lose much detail in the multi-layer structure of the network.

In terms of training speed, as shown in Table 1, the network inference speed will be slightly improved after the resolution is reduced, but this time difference is not enough for us to sacrifice part of the recovery effect, so 512 × 512 is still a better choice.

#### 4.2.2. Loss Function Selection

In terms of loss functions, L2 Loss and L1 Loss are the most commonly used regression loss functions in image restoration tasks [35]. The difference between them is mainly reflected in the attitude towards outliers. The former is sensitive to outliers and needs to be given more weight to predict and deal with outliers, but gives a more stable closed-form solution. The latter is more robust in dealing with outliers, but the gradient is constant and the solving efficiency is low.

**Figure 7:**
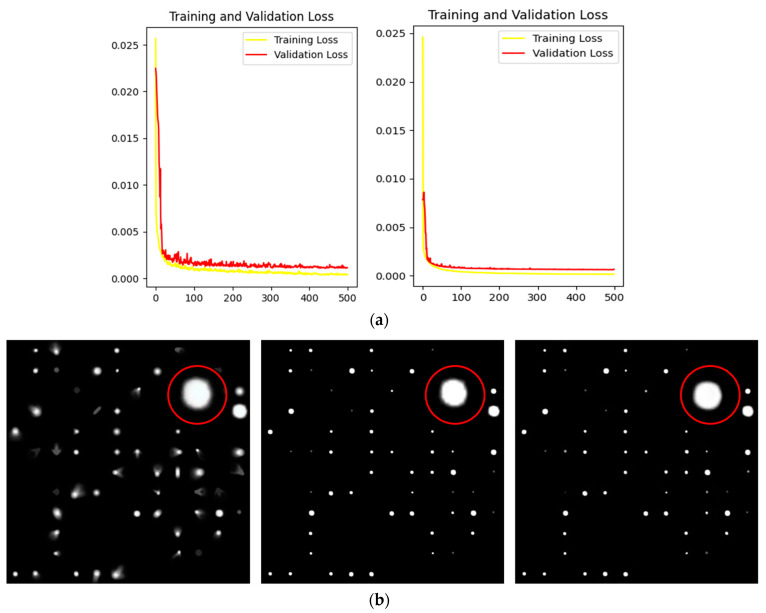
(**a**) Loss curves of 500 epochs were trained with L1 Loss and L2 Loss respectively; (**b**) the original drawing; L1 Loss was used to restore the image. Images recovered using L2 Loss.

In order to choose the better one from these two methods, we used L2 Loss and L1 Loss, respectively, to train 500 epochs of the network without changing all other parameters, in which the loss curve and image restoration effect are taken as reference items, as shown in Figure 7.

As can be seen from the loss curve in Figure 7, there is no obvious difference between the two in the training set. In the validation set, although the final convergence value of the two reached a good level, the curve of L2 Loss was significantly smoother. From the view of the restored image, the outer edge contour of the star point was significantly softer and smoother in the image restored using L2. Therefore, we finally selected L2 Loss as the loss function.

#### 4.2.3. Optimizer Selection

The optimizer has many options, such as SGD, Adgrad, and Adam, each of which has its own advantages and disadvantages.

Firstly, the overall optimization framework of the optimizer is introduced, in which the parameter to be optimized is written as w, the objective function is $f\left(w\right)$, and the learning rate is α:

For each epoch (t):

1. Calculate the gradient of the objective function with respect to parameters:

(15) $$g_{t}=\nabla f\left(w_{t}\right)$$

2. Calculate first-order momentum and second-order momentum according to the historical gradient:

(16) $$m_{t}=\phi (g_{1},g_{2},\ldots ,g_{t})$$

(17) $$V_{t}=\psi (g_{1},g_{2},\ldots ,g_{t})$$

3. Calculate the descending gradient at the current moment:

(18) $$\eta_{t}=\alpha •m_{t}/\sqrt{V_{t}}$$

In fact, the learning rate changed from α to $\alpha /\sqrt{V_{t}}$.

4. Update parameters according to the descending gradient:

(19) $$w_{t+1}=w-\eta_{t}$$

Steps 3 and 4 are consistent for each algorithm, and the main difference is reflected in 1 and 2.

As the most representative optimization method, SGD does not have the concept of momentum, that is to say:

(20) $$m_{t}=g_{t}$$

(21) $$V_{t}=I^{2}$$

and the descent gradient is $\eta_{t}=\alpha •g_{t}$. SGD tends to converge well, but the learning rate is constant, leads to a slow decline rate, and may oscillate continuously on both sides of the gully, staying at a local optimum.

The concept of second-order momentum was added to Adagrad, so that the learning rate could be adjusted adaptively. For parameters with more updates, the accumulated information could not be affected by a single sample, so the learning rate could be lower. For parameters with occasional updates, the learning rate needed to be higher in order to know more information. The second order momentum is represented by the sum of the squares of all gradients in that dimension:

(22) $$V_{t}=\sum_{\tau }^{t}g_{\tau }^{2}$$

This method is a greater improvement in the parameter space towards a more gentle tilt and is suitable for scenarios with sparse data. However, since the second-order momentum is one-way increasing, the learning rate continues to decline and the convergence rate gradually slows down. Once the learning rate decreases to 0, the learning process of the network stops, no matter whether there is still no learned data.

Adam is the most commonly used optimization method at present. At the same time, first-order vector and second-order vector introduce the optimization process, so there is:

(23) $$m_{t}=\beta_{1}•m_{t-1}+(1-\beta_{1})•g$$

(24) $$V_{t}=\beta_{2}*V_{t-1}+(1-\beta_{2})•g_{t}^{2}$$

These are also common $\beta_{1}$ and $\beta_{2}$ in the Adam code to manipulate first-order and second-order vectors, respectively.

As can be seen from the above two vector formulas, Adam is not sensitive to the learning rate, so it is often set to 0.0001 by default. Compared with Adagrad, Adam only accumulates second-order momentum within a fixed time window without storing all gradients globally, so it is suitable for large-scale data processing. However, Adam also depends more on time and parameter gradient. With the change of time, if there is a sudden change of data, it may cause a change in $V_{t}$, resulting in the shock of the learning rate in the later period, and the network cannot converge.

**Figure 8:**
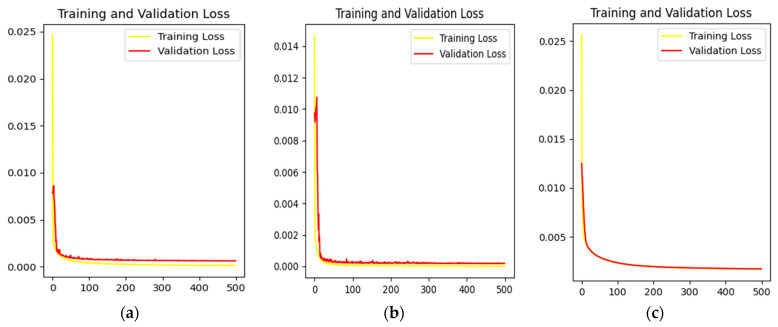
(**a**) Loss curve of training set and verification set when Adagrad is used as optimizer; (**b**) loss curve of training set and verification set when Adam is used as optimizer; (**c**) loss curve of training set and verification set when SGD is used as optimizer.

Each of the three methods has its pros and cons, and we trained each as an optimizer in order to choose the more appropriate one. The training data came from our simulation dataset, and the Loss function was L2 Loss, with a total of 500 epochs. The loss curve of the training process is shown in Figure 8.

As can be seen from Figure 8, SGD converged slowly in the early stage and the curve was smooth, but Adagrad and Adam converged quickly. However, the convergence curves of both of them showed different degrees of oscillation, and the stability was slightly poor. From a loss and accuracy point of view, Adagrad’s value was the best of the three optimizers due to the incomplete convergence of SGD.

**Figure 9:**
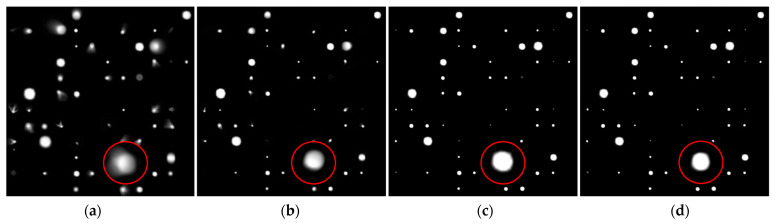
(**a**) Original images; (**b**) images restored when using SGD; (**c**) images restored when using Adagrad; (**d**) images restored when using Adagrad + SGD.

In order to better balance the learning speed and recovery effect of the network, we tried to use the combination of SGD and Adagrad: the first 300 EPOches adopted Adagrad, which had the advantage of fast convergence. After 200 epochs, we switched to SGD and slowly found the optimal solution. Similarly, we conducted 500 rounds of training on the same dataset using SGD, Adagrad, and their combinations, respectively. We then extracted an image from the dataset and tested its recovery, as shown in Figure 9.

In Figure 9b, due to incomplete convergence of SGD, the stars in the figure still had some residual aberrations, and some dim stars were not found. Figure 9d shows the test results of Adagrad as the optimizer. Compared with SGD, this method increased the number of stars and corrected optical aberration effectively. Figure 9d shows a reconstructed image combining the two optimizers. In addition to effective aberration correction, the outer edges of the stars were more fluxy-contouring, giving each star a greater concentration of energy.

Therefore, the combination of our task SGD and Adagrad was the best way to optimize.

### 4.3. Ablation Experiments

In this section, we perform ablation experiments. Our network structure was based on an encoder–decoder structure, referring to U-Net, whose internal structure was different from U-Net. Therefore, we divided the network into four parts: the ordinary encoder–decoder network (consisting only of convolution, upsampling, and downsampling), ASANet’s basic network (with the addition of GN + Leaky ReLU), skip connections, and a self-attention mechanism. Through ablation experiments, we gradually demonstrated that the structural design of ASANet made sense.

**Figure 10:**
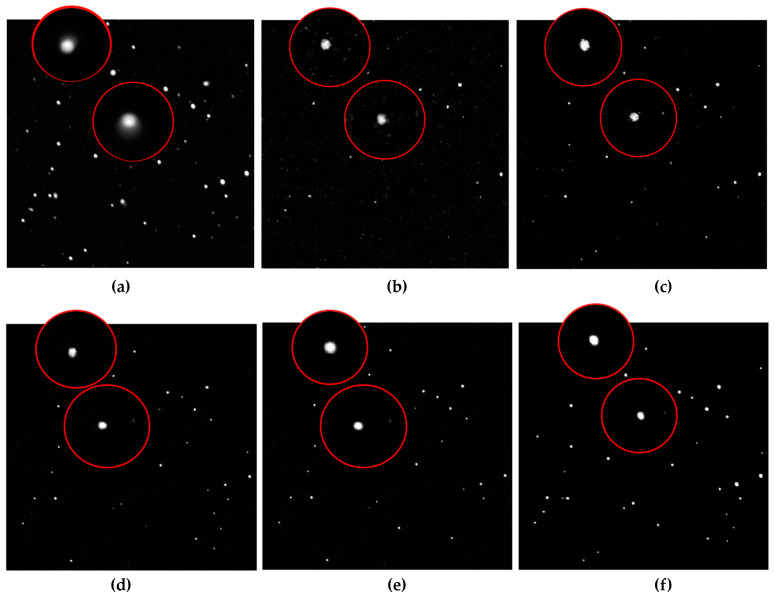
(**a**) The original image. (**b**) Images restored when using encoder–decoder; (**c**) images restored when using the basic network; (**d**) images restored when using ASAnet without self-attention; (**e**) images restored when using ASAnet without skip connection; (**f**) images restored when using ASANet.

In this section, we conducted five groups of experiments, which used the encoder–decoder mechanism, ASAnet infrastructure, ASANet without self-attention, ASAnet without skip connection, and ASAnet, respectively, to restore real images. The training strategy is described in Section 3.3. Because the source of the images is confidential, we will not discuss them here. The effect of image restoration was evaluated from subjective and objective perspectives. The subjective approach was to visually view the restored image, as shown in Figure 10. Objective methods are quality evaluation indicators, such as PSNR and SSIM, as shown in Table 2.

For an intuitive comparison, two locations were selected and the stars at the corresponding locations were enlarged in the six images in Figure 10, where Figure 10a is the original image. As can be seen from the circled area, the shape of the two recovered stars in Figure 10b was not clear, and the weak stars were largely lost. At the same time, due to the extreme lack of network learning ability, the reconstructed image even showed artifacts. Figure 10c removed the artifacts and retrieves a small amount of the missing star, but their energy is weaker, causing the star to dim. The star still did not have a regular shape, just two irregular clumps of pixels. In Figure 10d, only the basic structure +skip connection was used to retain the underlying features, so the number of stars increased significantly. Here the stars had a profile, and their energy was more concentrated. In Figure 10e, the basic structure +self-attention was used to better assign weight to the network, so the star had shrunk into a circle with concentrated energy. However, because many relatively faint and small stars were lost in the process of convolution and downsampling, the feature map sent to self-attention lacked underlying features. Although self-attention can capture the weight relationship of the entire image, the incomplete feature map made the number of star points still less than that in Figure 10d. Figure 10f was made using our ASANet. Although the star in this image was not perfectly round, its outline was smooth and natural, with concentrated energy. Almost all stars in other locations could be reconstructed, with good results.

As can be seen from the evaluation indexes in Table 2, with the improvement of the network structure, the quality evaluation of the restored image gradually increased. Due to differences in learning effectiveness, both the encoder–decoder structure and ASAnet’s infrastructure obtained low values, with the encoder–decoder structure obtaining the lowest value due to the presence of artifacts in the image. With the addition of jump connections or self-attention, the quality evaluation was raised to a new level, which also corresponded to the significant improvement of the recovery effect in Figure 9d,e. Of course, it was the images recovered using ASAnet that ended up with the highest scores.

Through the verification of Figure 10 and Table 2, it can be determined that each structure plays an important role in our network, which together enables our network to better recover astronomical images.

### 4.4. Comparison with Different Methods

Currently, in the field of deep learning, the most widely used image restoration network architectures include the deep automatic encoder (DAE), generative adduction network (GAN), and cascading network. The depth autoencoder first extracts the image features, and the decoder reconstructs the image according to these features. Our method belongs to this category. The GAN method tends to make the generator produce clear images, so that the discriminator cannot distinguish them from the real clear images. The cascaded network consists of several modules, which are cascaded successively to build a deeper structure and process blurred images in stages. The strategy of a multi-scale deblurring network is to first restore low-resolution deblurring images and then gradually generate clear results with high resolution.

Since cascaded networks usually utilize CNNs as fuzzy kernel estimators to construct a two-stage image deblurring framework, i.e., a CNN-based fuzzy kernel estimation stage and a kernel-based deconvolution stage, which belong to the early methods, while the current deep-learning methods aim to directly learn the complex relationship between image blur and clarity. Therefore, we chose three networks, corresponding to the remaining three types of methods mentioned above, for experimental comparison, which are the encoder–decoder structure with nested skip connections proposed by Gao et al. [36] (the authors did not name the network, so we will refer to it as NSC-ED in the following), DeblurGan proposed by Kupyn [37], and the multiscale deblurring method proposed by Nah et al. [16].

For NSC-ED, we processed the images as 256 × 256 as training inputs for a total of 3000 pairs. During training, the batch size was set to 8 and all weights were initialized using the Xavier method; the bias was initialized to 0. The network was optimized using the Adam method with default settings of $\beta_{1}$ = 0.9, $\beta_{2}$ = 0.999 and ε = 10^−8^. The learning rate was initially set to 0.0001 and decayed to 0 using a power exponent of 0.3.

For DeblurGan, a total of 1000 images with a resolution of 256 × 256 were input. The loss function was set to the sum of content loss and adversarial loss, using Adam for optimization. The learning rate was set initially to 0.0001 for both the generator and critic (discriminator). After the first 150 epochs, we linearly decayed the rate to zero over the next 150 epochs.

For multi-scale, we also used our dataset with 3000 pairs of images as the input. The loss function was a combination of multi-scale MSE loss and adversarial loss, using Adam as the optimizer, with a batch size = 2 and a learning rate of 0.00002, for a total of 900 iterations.

The above training strategy basically follows the original authors’ choice, but we made some minor changes to the batch size and the number of iterations due to the computational power limitation of the server and the reduced difficulty of image learning.

In addition, in the field of traditional methods, RL, as one of the most classical algorithms, is still applied flexibly in the image field and is the most common method in practical applications. In this paper, for realistic purposes, we chose to include the RL + Tikhonov regularization method in our experiments.

**Figure 11:**
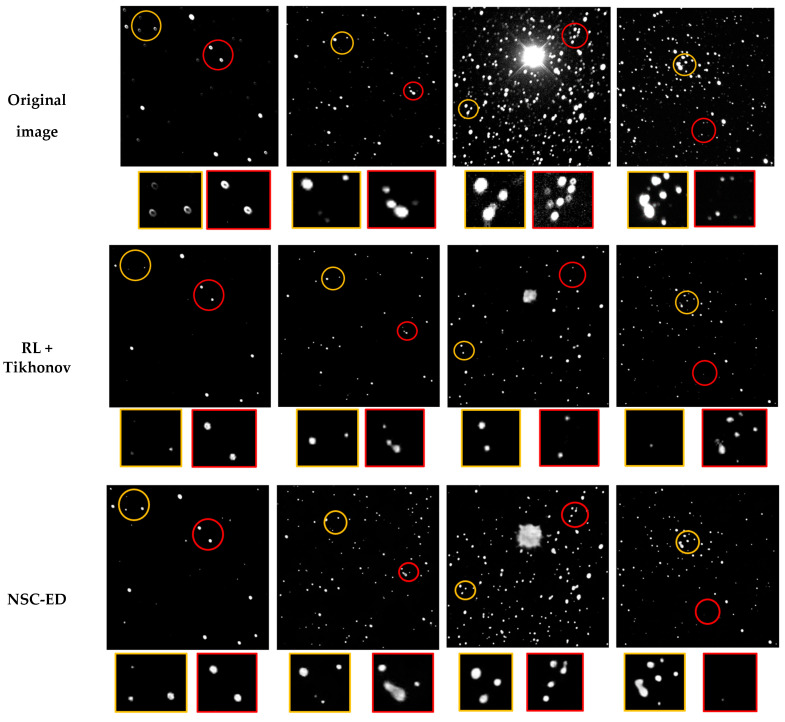

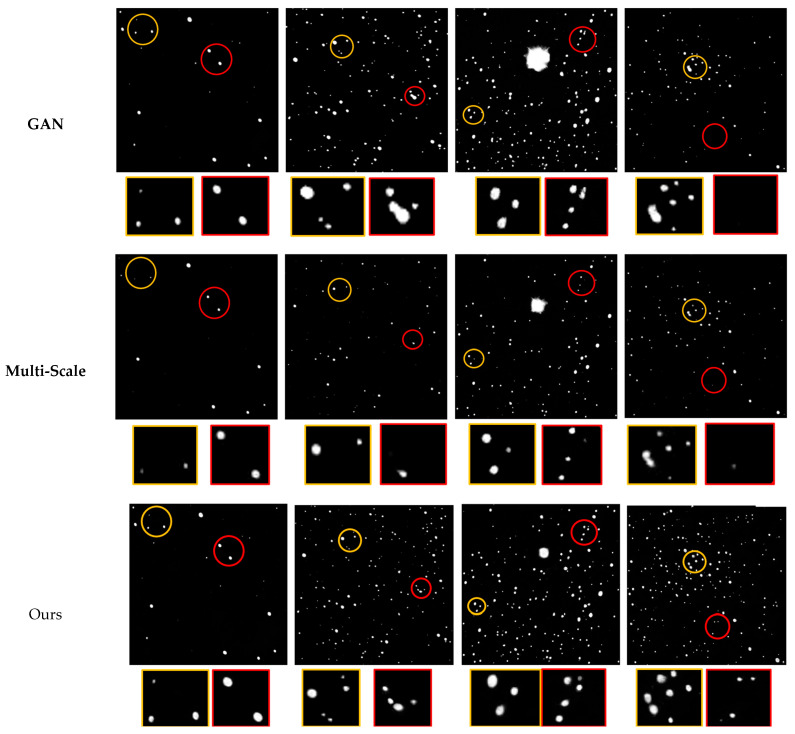
Use different methods to restore the results of real astronomical images. Each column represents a method, and each row inputs the same set of images.

In this round of experiments, we prepared several sets of real images and restored them separately using the above method. The restored images are shown in Figure 11, and we arranged them in vertical rows for easy viewing. For easier viewing, we zoomed in on the same parts selected from each set of images. The stars in these regions are either densely distributed or have significant optical aberrations, making it easy to compare the recovery effects.

According to the restoration results in Figure 11, it can be seen that RL, as a classical method, is not flexible enough to deal with PSF images with spatial variations. After many iterations, star points were lost, energy could not be collected, and the generated details were poor. Both NSC-ED and multiscale networks achieved image restoration by different levels and multiple processing, and the energy aggregation effect was better than RL, but the detailed features were still easily lost. Among them, NSC-ED can retain the underlying features better due to the presence of nested skip connection, so the number of star points recovered was more. Multi-scale uses multi-scale images as input, but the star map is very sensitive to the resolution, and the change of scale will lead to the loss of some information, so the number of stars recovered is not much. Compared with these two, the GAN network mechanism is simple, and the generated images can remember the star positions well and retain the details better, but there is some difference in the correction effect of distortion. Our network, on the other hand, has a skip connection, which can effectively preserve the underlying features and retain the number of stars better. The addition of self-attention enhances the feature extraction ability and is more friendly to recover the shape contour of stars.

From an objective point of view, we should also evaluate the quality of these images. We calculated the PSNR and SSIM of these images and took their average values recorded in Table 3.

The quality metrics in Table 3 can be used as the most direct criterion, except that the GAN-based model quality evaluation indexes are usually IS (inception score) and FID (Frechet inception distance), with poor performance in PSNR or SSIM. The index values of the other methods are all lower than our ASAet, which also proves that the effect of ASAnet is superior to other methods.

Combining the above comparison results, our proposed ASANet achieves better results than the other methods.

Of course, we do not exclude that the change of training strategy may lead to the performance degradation of other methods, which instead proves that our network can be trained with fewer data.

## 5. Discussion

The proposed ASANet method has a strong learning capability to more accurately restore astronomical images with spatially varying PSFs, and requires very little data to build a robust network model.

Compared with traditional methods, the end-to-end approach can reduce the reliance on a priori information and does not need to consider PSF modeling. Compared to other deep-learning methods, our approach uses a relatively simple network structure to achieve the restoration of single-frame astronomical images by introducing a self-attentive mechanism that takes into account both global details and local structure.

In addition, we do not intentionally deal with noise during pre-processing. After real image restoration, we found that this network actually has some denoising ability. This ability is unstable without training and needs to be investigated more deeply in subsequent studies.

## 6. Conclusions

In this paper, we proposed a method called ASANet to repair astronomical images.

First, we proposed a network called ASANet to accomplish the restoration of spatially variant astronomical images. In this network, the feature extraction capability was improved by changing hyperparameters such as normalization and adding skip connections. In addition, a self-attention module was added to make better use of the original feature layer when fusing different feature mapping information. This network structure allowed us to train a powerful network model with fewer data for the end-to-end restoration of astronomical images. In addition, astronomical image datasets with spatially varying PSFs were independently constructed for training.

Then, we conducted several experiments. To select an appropriate training strategy, we validated the input images with different resolutions and selected the most suitable loss function and optimizer for this study. To verify the necessity of improving the network structure, we conducted an ablation study to demonstrate that ASAnet can better fuse feature information at different levels and combine local and global feature distribution weights, which can help the network to acquire feature information of images more effectively and achieve image recovery.

Finally, we compared different methods, including three deep-learning methods and one traditional method, and verified that ASANet can better eliminate optical aberrations in images and achieve high-quality astronomical image restoration.

Unfortunately, the limited computational power of the server and the limited image resolution and dataset image size used for learning limited the actual restoration effect of the network to some extent. In addition, there was often some noise in real astronomical images, and although we did not specifically consider noise, ASANet showed the potential of de-noising in our experiments.

In the next research, we will further optimize the network structure, reduce the computational effort, and conduct more targeted research on noise reduction to ensure a more perfect result in applications.

## Figures and Tables

**Table 1:** The time to process an image of different resolutions.

Resolution	512 × 512	256 × 256
Time	63 ms	54 ms

**Table 2:** Quantitative results for different network architecture.

Description	PSNR	SSIM
Encoder–decoder	31.7839	0.8747
Basic network	32.1961	0.8803
Basic network + skip connection	34.5649	0.9012
Basic network + self-attention	34.6324	0.9034
ASANet	35.4375	0.9157

**Table 3:** Quantitative results of different methods.

	RL	NSC-ED	GAN	Multi-Scale	Ours
PSNR	34.5014	34.9134	34.4641	34.7096	35.4154
SSIM	0.8802	0.8981	0.8781	0.8901	0.9124

## Data Availability

Not applicable.

## References

[1] Tyson J.A., Wolff S. (2002). Survey and Other Telescope Technologies and Discoveries. Surv. Other Telesc. Technol. Discov..

[2] Ackermann M.R., McGraw J.T., Zimmer P.C. An Overview of Wide-Field-Of-View Optical Designs for Survey Telescopes. Proceedings of the Advanced Maui Optical and Space Surveillance Technologies Conference.

[3] Wilson R.N. (2004). Reflecting Telescope Optics I. Basic Design Theory and Its Historical Development.

[4] Jee M.J., Tyson J.A. (2011). Toward Precision LSST Weak-Lensing Measurement. I. Impacts of Atmospheric Turbulence and Optical Aberration. Publ. Astron. Soc. Pac..

[5] Jia P., Sun R., Wang W., Cai D., Liu H. (2017). Blind deconvolution with principal components analysis for wide-field and small-aperture telescope. Mon. Not. R. Astron. Soc..

[6] Ben Hadj S., Blanc-Féraud L., Aubert G. (2014). Space Variant Blind Image Restoration. SIAM J. Imaging Sci..

[7] Ciliegi P., La Camera A., Schreiber L., Bellazzini M., Bertero M., Boccacci P., Diolaiti E., Foppiani I., Lombini M., Massari D. Image restoration with spatially variable PSF. Proceedings of the SPIE Adaptive Optics Systems IV.

[8] Harmeling S., Hirsch M., Schölkopf B. (2010). Space-Variant Single-Image Blind Deconvolution for Removing Camera Shake. Advances in Neural Information Processing Systems (NIPS).

[9] Hirsch M., Sra S., Schölkopf B., Harmeling S. Efficient filter flow for space-variant multi-frame blind deconvolution. Proceedings of the IEEE Conference on Computer Vision and Pattern Recognition (CVPR).

[10] Nagy J.G., O’Leary D.P. (1998). Restoring images degraded by spatially variant blur. SIAM J. Sci. Comput..

[11] Schuler C.J., Hirsch M., Harmeling S., Schölkopf B. (2016). Learning to Deblur. IEEE Trans. Pattern Anal. Mach. Intell..

[12] Yan R., Shao L. (2016). Blind Image Blur Estimation via Deep Learning. IEEE Trans. Image Process..

[13] Sun J., Cao W., Xu Z., Ponce J. Learning a Convolutional Neural Network for Non-uniform Motion Blur Removal. Proceedings of the IEEE Conference on Computer Vision and Pattern Recognition (CVPR).

[14] Zhang J., Pan J., Ren J., Song Y., Bao L., Lau R.W., Yang M.H. Dynamic Scene Deblurring Using Spatially Variant Recurrent Neural Networks. Proceedings of the 2018 IEEE/CVF Conference on Computer Vision and Pattern Recognition.

[15] Yuan Y., Su W., Ma D. Efficient Dynamic Scene Deblurring Using Spatially Variant Deconvolution Network with Optical Flow Guided Training. Proceedings of the 2020 IEEE/CVF Conference on Computer Vision and Pattern Recognition (CVPR).

[16] Nah S., Hyun Kim T., Mu Lee K. Deep Multi-scale Convolutional Neural Network for Dynamic Scene Deblurring. Proceedings of the 2017 IEEE Conference on Computer Vision and Pattern Recognition (CVPR).

[17] Graham L., Yitzhaky Y. (2017). Blind restoration of space-variant Gaussian-like blurred images using regional PSFs. Signal Image Video Process..

[18] Jung H.M., Kim B.H., Kim M.Y. (2020). Residual forward-subtracted Ushaped network for dynamic and static image restoration. IEEE Access.

[19] Jin X., Hu Y., Zhang C.Y. Image restoration method based on GAN and multi-scale feature fusion. Proceedings of the 2020 Chinese Control and Decision Conference.

[20] Ramakrishnan S., Pachori S., Gangopadhyay A., Raman S. Deep Generative Filter for Motion Deblurring. Proceedings of the IEEE International Conference on Computer Vision Workshops CVPR.

[21] Flamary R. Astronomical image reconstruction with convolutional neural networks. Proceedings of the 2017 25th European Signal Processing Conference (EUSIPCO).

[22] Schawinski K., Zhang C., Zhang H., Fowler L., Santhanam G.K. (2017). Generative Adversarial Networks recover features in astrophysical images of galaxies beyond the deconvolution limit. Mon. Not. R. Astron. Soc. Lett..

[23] Domínguez Sánchez H., Huertas-Company M., Bernardi M., Tuccillo D., Fischer J.L. (2018). Improving galaxy morphologies for SDSS with Deep Learning. Mon. Not. R. Astron. Soc..

[24] Sureau F., Lechat A., Starck J.L. (2019). Deep learning for a space-variant deconvolution in galaxy surveys. Astron. Astrophys..

[25] Akhaury U., Starck J.L., Jablonka P., Courbin F., Michalewicz K. (2022). Deep learning-based galaxy image deconvolution. Front. Astron. Space Sci..

[26] Buncher B., Sharma A.N., Carrasco Kind M. (2021). Survey2Survey: A deep learning generative model approach for cross-survey image mapping. Mon. Not. R. Astron. Soc..

[27] Nammour F., Akhaury U., Girard J.N., Lanusse F., Sureau F., Ali C.B., Starck J.L. (2022). ShapeNet: Shape constraint for galaxy image deconvolution. Astron. Astrophys..

[28] Ronneberger O., Fischer P., Brox T. (2015). U-NET: Convolutional Networks for Biomedical Image Segmentation. Medical Image Computing and Computer-Assisted Intervention–MICCAI 2015: 18th International Conference, Munich, Germany, 5–9 October 2015, Proceedings, Part III 18.

[29] Ibtehaz N., Rahman M.S. (2020). MultiResU-Net: Rethinking the U-Net architecture for multimodal biomedical image segmentation. Neural Netw..

[30] Lou A., Guan S., Loew M. (2021). DC-U-Net: Rethinking the U-Net architecture with dual channel efficient CNN for medical image segmentation. Proceedings of the Medical Imaging 2021: Image Processing.

[31] Dong H., Pan J., Xiang L., Hu Z., Zhang X., Wang F., Yang M.H. Multi-Scale Boosted Dehazing Network with Dense Feature Fusion. Proceedings of the IEEE/CVF Conference on Computer Vision and Pattern Recognition (CVPR), 2020.

[32] Cho S.J., Ji S.W., Hong J.P., Jung S.W., Ko S.J. Rethinking Coarse-to-Fine Approach in Single Image Deblurring. Proceedings of the 2021 IEEE/CVF International Conference on Computer Vision (ICCV).

[33] Wang X., Girshick R., Gupta A., He K. Non-local Neural Networks. Proceedings of the IEEE/CVF Conference on Computer Vision and Pattern Recognition.

[34] CANDELS—Cosmic Assembly Near-infrared Deep Extragalactic Legacy Survey. http://arcoiris.ucolick.org/candels/.

[35] Zhao H., Gallo O., Frosio I., Kautz J. (2017). Loss Functions for Image Restoration with Neural Networks. IEEE Trans. Comput. Imaging.

[36] Gao H., Tao X., Shen X., Jia J. Dynamic Scene Deblurring with Parameter Selective Sharing and Nested Skip Connections. Proceedings of the 2019 IEEE/CVF Conference on Computer Vision and Pattern Recognition (CVPR).

[37] Kupyn O., Budzan V., Mykhailych M., Mishkin D., Matas J. Deblurgan: Blind motion deblurring using conditional adversarial networks. Proceedings of the 2018 IEEE/CVF Conference on Computer Vision and Pattern Recognition.

